# Severe acute respiratory syndrome coronavirus 2 (SARS-CoV-2) antibody lateral flow assay for antibody prevalence studies following vaccination: a diagnostic accuracy study

**DOI:** 10.12688/wellcomeopenres.17231.2

**Published:** 2022-05-26

**Authors:** Alexandra Cann, Candice Clarke, Jonathan Brown, Tina Thomson, Maria Prendecki, Maya Moshe, Anjna Badhan, Bryony Simmons, Bob Klaber, Paul Elliott, Ara Darzi, Steven Riley, Deborah Ashby, Paul Martin, Sarah Gleeson, Michelle Willicombe, Peter Kelleher, Helen Ward, Wendy S. Barclay, Graham S. Cooke

**Affiliations:** 1 Department of Infectious Disease, Imperial College London, London, UK; 2 Imperial College Healthcare NHS Trust, London, UK; 3 Centre for Inflammatory Disease, Department of Immunology and Inflammation, Imperial College London, London, UK; 4 Imperial College Renal and Transplant Centre, Imperial College Healthcare NHS Trust, London, UK; 5 LSE Health, London School of Economics and Political Science, London, UK; 6 School of Public Health, Imperial College London, London, UK; 7 MRC Centre for Global infectious Disease Analysis and Abdul Latif Jameel Institute for Disease and Emergency Analytics, Imperial College London, London, UK; 8 National Institute for Health Research, Imperial Biomedical Research Centre, London, UK; 9 Chelsea & Westminster NHS Trust, London, UK

**Keywords:** SARS-CoV-2, Covid-19, Lateral flow, LFIA, Antibodies, Neutralisation, Seroprevalence

## Abstract

**Background:** Lateral flow immunoassays (LFIAs) are able to achieve affordable, large scale antibody testing and provide rapid results without the support of central laboratories. As part of the development of the REACT programme extensive evaluation of LFIA performance was undertaken with individuals following natural infection. Here we assess the performance of the selected LFIA to detect antibody responses in individuals who have received at least one dose of severe acute respiratory syndrome coronavirus 2 (SARS-CoV-2) vaccine.

**Methods:** This was a prospective diagnostic accuracy study. Sampling was carried out at renal outpatient clinic and healthcare worker testing sites at Imperial College London NHS Trust. Two cohorts of patients were recruited; the first was a cohort of 108 renal transplant patients attending clinic following two doses of SARS-CoV-2 vaccine, the second cohort comprised 40 healthcare workers attending for first SARS-CoV-2 vaccination and subsequent follow up. During the participants visit, finger-prick blood samples were analysed on LFIA device, while paired venous sampling was sent for serological assessment of antibodies to the spike protein (anti-S) antibodies. Anti-S IgG was detected using the Abbott Architect SARS-CoV-2 IgG Quant II CMIA. A total of 186 paired samples were collected. The accuracy of Fortress LFIA in detecting IgG antibodies to SARS-CoV-2 compared to anti-spike protein detection on Abbott Assay

**Results:** The LFIA had an estimated sensitivity of 92.0% (114/124; 95% confidence interval [CI] 85.7% to 96.1%) and specificity of 93.6% (58/62; 95% CI 84.3% to 98.2%) using the Abbott assay as reference standard (using the threshold for positivity of 7.10 BAU/ml)

**Conclusions:** Fortress LFIA performs well in the detection of antibody responses for intended purpose of population level surveillance but does not meet criteria for individual testing.

## Introduction

As vaccination programmes for coronavirus disease 2019 (COVID-19) are rolled out worldwide, population antibody testing is useful in monitoring immune responses to vaccinations, informing discussion and decisions about booster doses, and assessing levels of potential protective immunity in the population ^1^.

Lateral flow immunoassays (LFIAs) have the potential to deliver affordable, large-scale testing of individuals and provide rapid results without the support of central laboratories Antigen lateral flow testing is already being used widely. This approach, using antibody lateral flow devices, has been used across England in the REACT2 (REal time Assessment of Community Transmission) ^2^ study to estimate the number of infections during the first wave of the COVID-19 pandemic ^2^, monitor the decline in antibody positivity over time ^3^ and assess population antibody prevalence following vaccine roll-out, most recently in Round 5 of the study published in February 2021 ^4^.

Prior to the scale up of antibody testing for surveillance, extensive clinical and laboratory evaluation of diagnostic accuracy following natural infection was performed on a range of LFIA antibody tests ^5, 6^, identifying one for subsequent use. The test selected (Fortress, Northern Ireland) detects antibody against the spike protein of the virus (contained in all licensed vaccines) and would therefore be expected to detect vaccine induced antibody responses. This study examined the accuracy of the Fortress LFIA device in detecting antibodies in two cohorts of vaccinated individuals and explored the relationship between LFIA results and viral neutralisation.

## Methods

This was a prospective diagnostic accuracy study conducted between 20th December 2020 and 26 ^th^ May 2021. Samples were collected from two groups: renal transplant patients (cohort 1) and healthcare workers (cohort 2).

### Bias

Every attempt was made to address potential sources of bias. All eligible participants were offered enrolment where practical and every effort was made to ensure understanding of the participant information sheet (PIS) and study procedure, using translation services where necessary. Potential participants were given time to consider participation and trained research staff were able to answer questions relating to the study.

### Eligibility criteria

Eligibility for both cohorts was defined as:

1) Adult (>/=18 years old)
2) Able to understand and consent to study
3) Received either one or two doses of any UK approved vaccine for COVID-19
4) Able to comply with study procedure/ study not thought to be risk to patient

### Sample size

Sample size was computed based on an expected sensitivity of 90% and specificity of 95%, with a minimal acceptable lower confidence limit of -10% for both estimates. Under power 1 – β = 0.85 and α = 0.05, the minimum **sample size** required is 106 cases and 76 controls. Patients were pragmatically enrolled to ensure minimum sample size achieved.

### Cohort 1: Renal transplant cohort

***Participants.*** Participants were recruited between 1 ^st^ February 2021 and 26 ^th^ May 2021.Those recruited were 108 renal transplant recipients who were attending clinic at Hammersmith Hospital 28 days (allowing range from 21 to 42 days) following a second dose of a SARS-CoV-2 vaccine, (either BNT162b2, Pfizer/BioNTech or ChAdOx1, Oxford/AstraZeneca). Participants were recruited directly from clinic by trained medical and nursing staff who explained the study and provided with PIS and informed consent form (ICF). Participants underwent a finger-prick capillary blood draw for immediate testing on the LFIA device and, at the same time, gave a venous blood samples for later serology testing. Clinical characteristics were obtained from electronic medical records (including basic demographic data, past medical history, vaccination status; see Table 1 for full details). All patients provided written informed consent. 

**Table 1.** Clinical characteristics renal transplant cohort. *Note table refers to Abbott serology result. Subsequently, one seronegative individual tested positive using DABA*.

Characteristic		All patients N=108 (%)	Anti-S Seronegative* N= 36 (%)	Anti-S Seropositive* N= 72 (%)
**Gender**	Male Female	74 (68.5) 34 (31.5)	25 (69.4) 11 (30.6)	49 (68.1) 23 (31.9)
**Age**	Years (Range)	54 (41–65)	44 (38–74)	56 (44–64)
**Ethnicity**	White Black Asian Other	52 (48.1) 8 (7.4) 34 (31.5) 14 (13.0)	17 (47.2) 2 (5.6) 11 (30.6) 6 (16.7)	35 (48.6) 6 (8.3) 23 (31.9) 8 (11.1)
**Cause of End Stage Kidney Disease**	Polycystic kidney disease Glomerulonephritis Diabetic nephropathy Urological Unknown Other	9 (8.3) 41 (38.0) 18 (16.7) 7 (6.5) 26 (24.1) 7 (6.5)	4 (11.1) 12 (33.3) 7 (19.4) 2 (5.6) 8 (22.2) 3 (8.3)	5 (6.9) 29 (40.3) 11 (15.3) 5 (6.9) 18 (25.0) 4 (5.6)
**Vaccinated ≤1 year post transplant**	Yes No	6 (5.6) 102 (94.4)	1 (2.8) 35 (97.2)	5 (6.9) 67 (93.1)
**Time vaccinated post-transplant**	Years (Median)	6.3 (2.9–11.9)	5.7 (2.8–11.7)	6.5 (2.9–12.0)
**Diabetes**	No Yes	75 (69.4) 33 (30.6)	27 (75.0) 9 (25.0)	48 (66.7) 24 (33.3)
**Vaccine type**	BNT162b2 ChAdOx1	51 (47.2) 57 (52.8)	12 (33.3) 24 (66.7)	39 (54.2) 33 (45.8)
**Time between vaccinations**	Days (median)	77 (73–80)	77 (71–80)	77 (74–80)
**Time of serological test post-boost**	Days (median)	34 (29–38)	34 (29–36)	34 (30–38)
**Prior COVID-19 exposure**	No Yes	89 (82.4) 19 (17.6)	36 (100.0) -	53 (73.6) 19 (26.4)

***Lateral flow immunoassay testing.*** Participants were supplied with an LFIA testing kit as used in the REACT home testing programme ^7^. The LFIA (Fortress, NI) detects IgG and IgM to the S1 subunit of the spike protein. Participants were also provided with verbal instructions on how to use the test by a member of the research team, prior to performing self-testing, with support provided where necessary. The LFIA result was assessed independently by two observers. The results were reported by the colour intensity of the IgG band, and documented as either a positive or negative result. 

### Cohort 2: Healthcare worker cohort

***Participants.*** Participants were recruited between 20 ^th^ December 2020 and 31 ^st^ January 2021. Overall, 39 healthcare workers were consented when attending for first vaccination with BNT162b2 Pfizer-BioNTech. Participants were approached by trained members of the research team at the vaccination centre and provided with a PIS and ICF with explanation of the study. Of these participants, 38 had repeat samples taken at 21 days post vaccination and one further participant had samples taken at 21 days who had not had day 0 samples. In total there were 40 participants. Data was collected on age and gender. Medical records of participants were not accessed for this cohort.

***Lateral flow immunoassay testing.*** Participants were supported in capillary blood draw for use with the Fortress LFIA devices as described above. Results were reviewed and recorded by the study team.

***Serological testing.*** Serological assessment was performed on the Abbott Architect SARS-CoV-2 IgG Quant II CMIA which reports a quantitative anti-Spike antibody titre. This assay has previously been reported to have specificity of 100% on stored pre-covid serum samples and sensitivity of 93.9% for samples ≥14 days post symptom onset ^8^. The threshold value for positivity of 7.10 binding antibody units (BAU)/ml. At the time of the study in the healthcare worker cohort (cohort 2), quantitative antibody titres were reported in AU/ml. To allow combination with cohort 1 data, these were converted to BAU/ml by multiplying by 0.142. Double antigen binding assay (DABA) testing for discordant results (positive LFIA with negative serological) was performed on available stored samples from cohort 1. Detailed methodology of DABA has been described previously. Briefly, the Imperial Hybrid DABA is a sequential two step double binding assay for the detection and measurement of antibody directed to the receptor binding domain of SARS-CoV-2. The proteins employed were expressed and gifted by the Crick Institute, London. In order to evaluate specificity the Hybrid DABA was tested on stored plasma and serum samples predating the SARS-CoV-2 outbreak (n=825) in which 0 samples tested positive, giving a specificity of 100%.

In addition, for cohort 2, individuals provided blood for assessment of neutralisation assays. The ability of sera to neutralise the SARS-CoV-2 virus was assessed by neutralisation assay on Vero cells. Sera were serially diluted in OptiPRO SFM (Life Technologies) and incubated for 1h at room temperature with 100 TCID50/well of SARS-CoV-2/England/IC19/2020 and transferred to 96-well plates pre-seeded with Vero-E6 cells. Serum dilutions were performed in duplicate. Plates were incubated at 37°C, 5% CO _2_ for 42 h before fixing cells in 4% PFA (paraformaldehyde). Cells were treated with methanol 0.6% H _2_O _2_ and stained for 1h with a 1:3000 dilution of 40143-R019 rabbit mAb to SARS-CoV-2 nucleocapsid protein (Sino Biological). A 1:3000 dilution of sheep anti-rabbit HRP (horseradish peroxidase) conjugate (Sigma) was then added for 1 h. TMB (3,3′,5,5′-Tetramethylbenzidine) substrate (Europa Bioproducts) was added and developed for 20 mins before stopping the reaction with 1M hydrogen chloride (HCl). Plates were read at 450nm and 620nm and the concentration of serum needed to reduce virus signal by 50% was calculated to give NT50 values.

### Performance analysis

The primary outcome of the study was sensitivity and specificity of the LFIA device in detecting SARS-CoV-2 IgG antibodies identified by the Abbott platform.

A secondary analysis was conducted using reference standard as either Abbott or, for discordant results (positive LFIA negative serology) in cohort 1 using in house DABA as reference standard for serological positivity.

Outcomes are presented with the corresponding binomial exact 95% confidence interval (95% CI). Statistical analyses (specificity and sensitivity) were performed with open access online website MedCalc diagnostic test evaluation calculator (version 20.015). Graphical analyses was performed using GraphPad Prism 9.1.2 software. An open-source alternative is R.

### Ethics approval

Ethics approvals were sought for each cohort prior to commencement of the study.

The renal cohort ethics were obtained from Health Research Authority, Research Ethics Committee (Reference: 20/WA/0123 - The Impact of COVID-19 on Patients with Renal disease and Immunosuppressed Patient).

For the healthcare worker cohort, we got ethics from the South Central-Berkshire B Research Ethics Committee (IRAS ID: 283805), and Medicines and Healthcare products Regulatory Agency approval for use of the LFIA for research purposes only.

## Results

### Cohort characteristics

The characteristics of the participants are described in Table 1 and Table 2. In total, 186 samples were tested using both LFIA and serological testing ^9^.

**Table 2.** Clinical characteristics of healthcare worker cohort.

Characteristic		All patients N=40 (%)	Anti-S Seronegative		Anti-S Seropositive	
			D0 N=26 (%)	D21 N=0 (%)	D0 N=13 (%)	D21 N=40 (%)
**Gender**	Male Female	13 (32.5) 27 (67.5)	10 (38.5) 16 (61.5)	0 0	3 (23.0) 10 (77.0)	13 (33.3) 27 (66.6)
**Age**	Years average (range)	43 (23–71)	46	-	38	43
**Vaccine type**	BNT162b2	40 (100)	- - - -			
**Time between vaccinations**	Days average (range)	65 (57–92)				

### LFIA IgG positivity and antibody titres in serum

**Figure 1.**
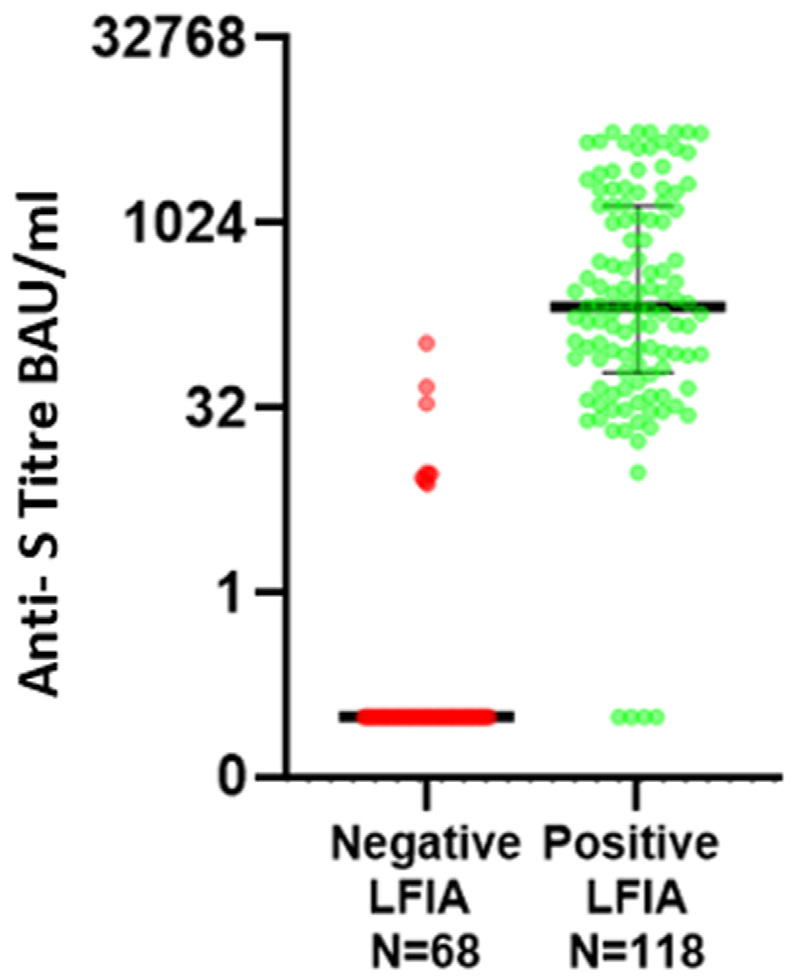
Paired Quantitative anti-S titres (Abbott) between those testing positive and negative on Fortress lateral flow immuno-assay (LFIA) for combined cohorts 1 and 2. (Y-axis uses Log 2 scale, data points below the lower limit of detection are assigned arbitrary value of 0.01 for plotting).

The combined results describe both cohort 1 and cohort 2 (n=186 samples, Figure 1). Of those samples which scored positive on LFIA (n=118), 4 had undetectable laboratory anti-S levels using Abbott Architect assay. Three of these samples (from the renal transplant cohort) were subsequently re-tested using an in-house DABA which detected antibodies in 1 sample and confirmed negativity in 2. The remaining 114 samples had a median anti-S titre of 229.5 BAU/ml and mean of 229.5 BAU/ml; anti-S titre ranged from 9.7 BAU/ml to 5680 BAU/ml. Of those which scored negative on LFIA (n=68), anti-S antibodies were detected in 10 samples, of which 7 had anti-S titre levels <10 BAU/ml (7.8, 8.0, 8.5, 8.8, 9.2, 9.4, 9.7). The other 58 negative LFIA tests had undetectable anti-S levels (<7.1 BAU/ml).

### Test sensitivity and specificity

***Primary analysis (Abbott as reference standard).*** Using the threshold value for positivity on serological testing of ≥7.10 BAU/ml, the LFIA had an estimated sensitivity of 92.0% (114/124; 95% CI 85.7% to 96.1%) and specificity of 93.6% (58/62; 95% CI 84.3% to 98.2%) using the Abbott assay as reference standard.

**Figure 2.**
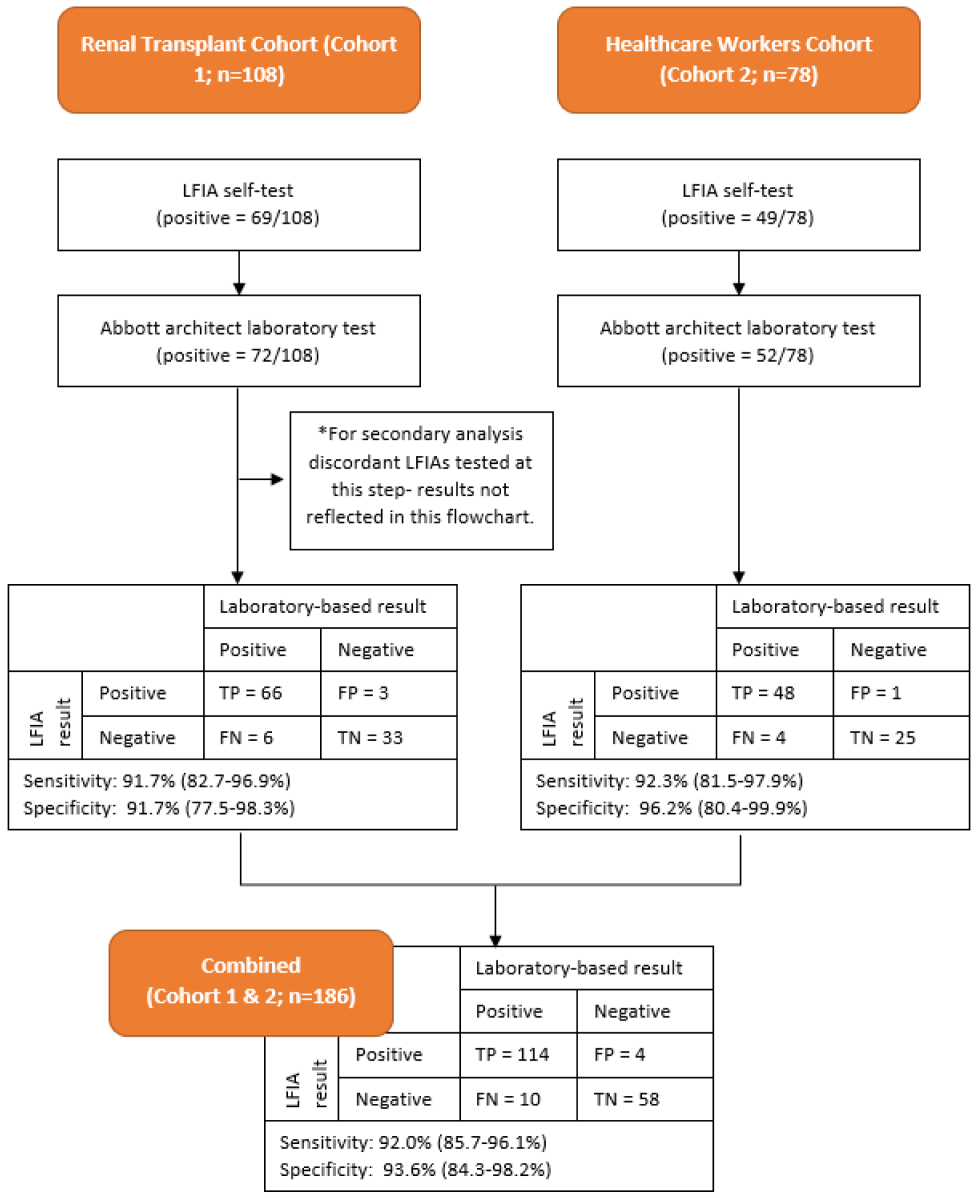
Flowchart detailing testing results for Cohort 1 and Cohort 2 and Combined Cohort. LFIA=Fortress lateral flow immuno-assay.

***Secondary analysis (Abbott or DABA as reference standard).*** Using the threshold vale for positivity on serological testing of ≥7.10 BAU/ml (n=183) on Abbott Architect Assay and confirmatory DABA result for available discordant samples (n=3) as the reference standard, the overall performance of the test in these combined cohorts produce an estimate of sensitivity of 92.0% (115/125; 95% CI 85.8% to 96.1%) and specificity of 95.1% (58/61; 95% CI 86.3% to 99.0%). Results were similar when analysing cohort 1 and cohort 2 individually (see Figure 2).

### Live virus neutralisation

**Figure 3.**
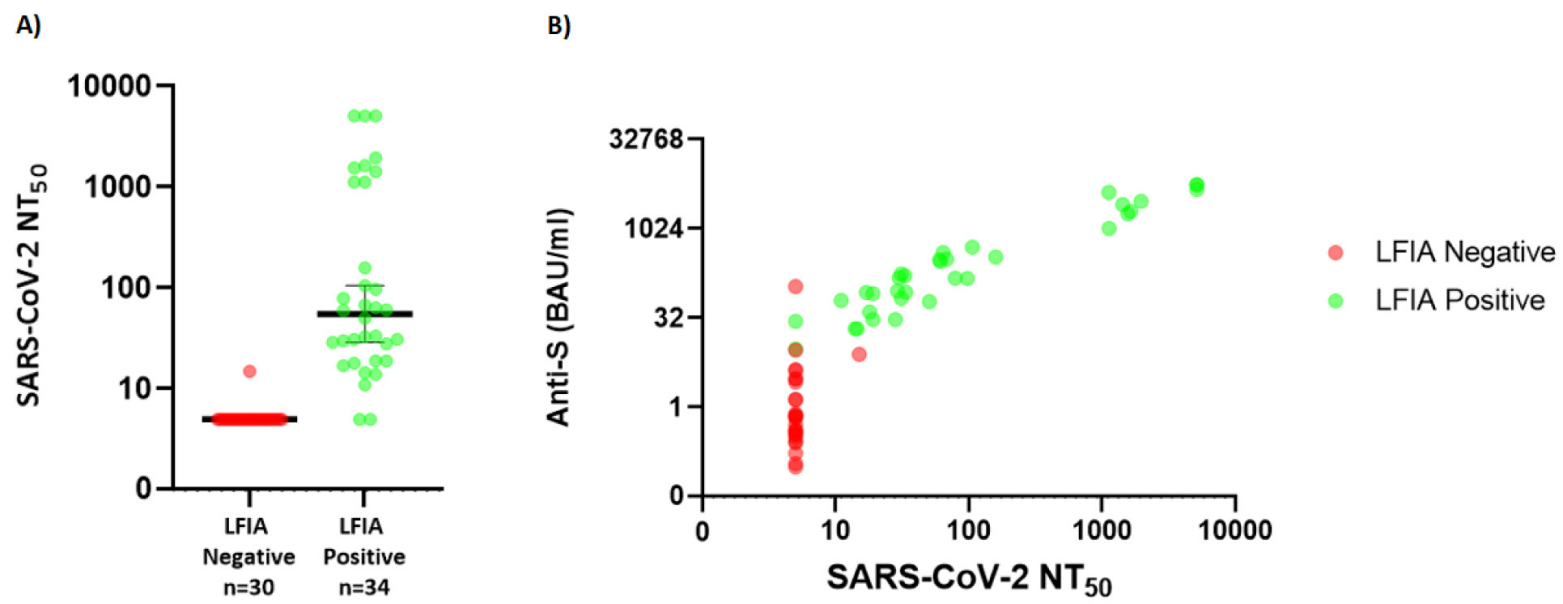
( **A**) Distribution of NT50 (neutralisation titre) values for individuals within cohort 2 according to whether positive or negative by Fortress lateral flow immuno-assay (LFIA) (Y-axis uses Log 10 scale, data points below the lower limit of detection are assigned arbitrary value of 0.01 for plotting) ( **B**) Relationship between neutralisation titre and anti-S titre in Abbott assay (green representing those LFIA positive, red LFIA negative). (Y-axis uses Log 2 scale, data points below the lower limit of detection are assigned arbitrary value of 0.01 for plotting). SARS-CoV-2 NT=severe acute respiratory syndrome coronavirus 2 neutralisation titre.

Neutralisation titres were available for 64/78 samples in the healthcare worker cohort. Neutralisation titres (NT50) were significantly higher in those with positive LFIA compared to those without ( Figure 3a). Only one LFIA-negative sample had detectable neutralisation assay using a threshold for positivity of (NT50 of 15 with an anti-S antibody titre of 7.8 BAU/ml). For individuals with detectable IgG on LFIA only 2/34 did not have significant evidence of viral neutralisation.

## Discussion

This study demonstrates that the Fortress LFIA device performs well in detecting IgG antibodies in vaccinated individuals when comparing against a serological assay widely used in routine practice. LFIAs have been a helpful tool the assessment of population antibody prevalence of SARS-CoV-2, and can play a role in informing vaccination strategy going forwards. The Fortress LFIA has been assessed previously for its performance following natural infection ^6^, though did not meet Medicines and Healthcare products Regulatory Agency (MHRA) criteria for individual use which recommend antibody tests should have a sensitivity of >98% (95% CI 96% to 100%) and specificity of >98% on a minimum of 200 known negative controls ^10^. The test has undergone extensive evaluation for home self-testing ^7^ and has since been used widely in community studies of antibody prevalence in England.

The performance of the LFIA in the cohorts of vaccinated individuals here demonstrates slightly higher sensitivity than previously reported for natural infection, though this difference is not significant. This is likely to reflect higher background titres of antibody following vaccination, particularly after second doses, when compared to natural infection in the community, at least in the healthcare worker cohort. The LFIA device does not detect very low levels of antibody which may still correlate with protection from severe disease and/ or hospitalisation. However, in the general population, the number of such individuals with low titres following two vaccinations will be low (in contrast to the renal transplant cohort studied here).

A small number of LFIA tests appear to produce false positive results (n=4) with undetectable antibodies in the commercial laboratory assay. To understand whether these were genuine false positives, these four samples were tested with a second sensitive assay (DABA). Only one if these discordant samples tested positive. 

There is growing evidence that the presence of neutralising antibodies in sera is highly predictive of protection from symptomatic COVID-19 disease ^11, 12^. Although the LFIA studied has a threshold below which it can’t detect Spike specific antibody that is present, that threshold is close to the level at which neutralising antibody can be reliably measured ( Figure 3a). This suggests that antibody positivity on the LFIA may give some indication of protection from symptomatic disease and thus could be useful to measure any waning of vaccine induced immunity in different populations.

The study has some limitations. The LFIAs were self-tested in the clinic or vaccination centre, where participants had access to support from trained healthcare professionals when required. This study does not fully replicate the ‘real-world’ application of LFIAs where users will be following a detailed guide in their own homes. Furthermore, the patient cohort includes healthcare workers and as such may have greater understanding and/or experience of self-testing than members of the general population. For this reason (and due to the relatively small sample size of this study with wide confidence intervals) there is a place for further studies with larger sample sizes in the community.

As a result of this pragmatic study design, some participants (13) in the HCW cohort provided two samples, at different time points, which were analysed within the study. As it is likely that there are differences in both the quantitative and qualitative immune responses in these samples, we analysed these samples independently given the primary purpose of the study was to evaluate the diagnostic accuracy of the LFIA compared to gold. If the 13 participants who provided two samples were removed from the calculations, the estimated sensitivity of the LFIA would be 87.7% with a wider (95%-CI 75.8- 97.1) for HCW cohort and to 91% (95%-CI 84.1- 95.6) overall.

The performance of the LFIA evaluated is sufficiently good that it can continue to play a helpful role in the assessment of population antibody responses resulting from widespread infection and high levels vaccination coverage, particularly given the correlation of LFIA results with the functional measure of live virus neutralisation. Over time, antibody titres will begin to wane and ongoing population surveillance can play a helpful role in informing decisions on policy for subsequent vaccination programmes, the targeting of booster vaccines. Rapid antibody testing may prove useful in initial screening of patients to receive monoclonal antibody therapy as lab methods may cause a delay in therapy to potentially eligible patients.

## Data availability

### Underlying data

Harvard Dataverse Repository: "Replication Data for: SARS-CoV-2 Antibody Lateral Flow Assay for Possible Use in Seroprevalence Surveys: a Diagnostic Accuracy Study", https://doi.org/10.7910/DVN/KCDZIN ^9^.

This project contains the following underlying data:

- Anti Spike Protein LFIA For HCW Cohort.tab

- Anti Spike Protein LFIA For Renal Cohort.tab

- Neutralisation Titres for HCW Cohort.tab

- DABA testing of LFIA Positive Abbott Negative Renal Transplant Samples.tab

Data are available under the terms of the Creative Commons Zero "No rights reserved" data waiver (CC0 1.0 Public domain dedication).

## References

[1] Maple PAC : Population (Antibody) Testing for COVID-19—Technical Challenges, Application and Relevance, an English Perspective. *Vaccines (Basel).* 2021;9(6):550. 10.3390/vaccines9060550 34073985 PMC8225097

[2] Ward H Atchison C Whitaker M : Antibody prevalence for SARS-CoV-2 following the peak of the pandemic in England: REACT2 study in 100,000 adults. *bioRxiv.* 2020. 10.1101/2020.08.12.20173690

[3] Ward H Cooke G Atchison C : Declining prevalence of antibody positivity to SARS-CoV-2: a community study of 365,000 adults.Cold Spring Harbor Laboratory. *medrxiv.* 2020. 10.1101/2020.10.26.20219725

[4] Ward H Cooke G Whitaker M : REACT-2 Round 5: increasing prevalence of SARS-CoV-2 antibodies demonstrate impact of the second wave and of vaccine roll-out in England.Cold Spring Harbor Laboratory. *medrxiv.* 2021. 10.1101/2021.02.26.21252512

[5] Flower B Brown JC Simmons B : Clinical and laboratory evaluation of SARS-CoV-2 lateral flow assays for use in a national COVID-19 seroprevalence survey. *Thorax.* 2020;75(12):1082–1088. 10.1136/thoraxjnl-2020-215732 32796119 PMC7430184

[6] Moshe M Daunt A Flower B : SARS-CoV-2 lateral flow assays for possible use in national covid-19 seroprevalence surveys (React 2): diagnostic accuracy study. *BMJ.* 2021;372:n423. 10.1136/bmj.n423 33653694 PMC7921617

[7] Atchison C Pristerà P Cooper E : Usability and Acceptability of Home-based Self-testing for Severe Acute Respiratory Syndrome Coronavirus 2 (SARS-CoV-2) Antibodies for Population Surveillance. *Clin Infect Dis.* 2021;72(9):e384–e393. 10.1093/cid/ciaa1178 32785665 PMC7454392

[8] Public Health England: Evaluation of the Abbott SARS-CoV-2 IgG for the detection of anti-SARSCoV-2 antibodies. [Accessed 20.5.22]. Reference Source

[9] Cann A : Replication Data for: SARS-CoV-2 Antibody Lateral Flow Assay for Possible Use in Seroprevalence Surveys: a Diagnostic Accuracy Study.Harvard Dataverse, V1. UNF:6:VAXtmGNrXcnYiw/LspmAzQ== [fileUNF]. 2021. 10.7910/DVN/KCDZIN

[10] MHRA: Target product profile antibody tests to help determine if people have immunity to SARS-CoV-2. [Accessed 21.6.21]. Reference Source

[11] Khoury DS Cromer D Reynaldi A : Neutralizing antibody levels are highly predictive of immune protection from symptomatic SARS-CoV-2 infection. *Nat Med.* Springer Science and Business Media LLC;2021;27(7):1205–1211. 10.1038/s41591-021-01377-8 34002089

[12] Earle KA Ambrosino DM Fiore-Gartland A : Evidence for antibody as a protective correlate for COVID-19 vaccines. *Vaccine.* Cold Spring Harbor Laboratory;2021;39(32):4423–4428. 10.1016/j.vaccine.2021.05.063 34210573 PMC8142841

