# Supplementary material for: Severe acute respiratory syndrome coronavirus 2 (SARS-CoV-2) antibody lateral flow assay for antibody prevalence studies following vaccination: a diagnostic accuracy study
Source: Wellcome Open Res. 2022 May 26;6:358. Originally published 2021 Dec 21. [Version 2] doi: 10.12688/wellcomeopenres.17231.2 (PMC9152464; doi:10.12688/wellcomeopenres.17231.2)
Supplement: Peer review history

# Peer review: Reviewer response for version 2

**DOI:** 10.21956/wellcomeopenres.19842.r50785

**Published:** 2022-05-30

**Authors:** Hans-Michael Kaltenbach

**Affiliations:** 1 Department of Biosystems Science and Engineering, Swiss Institute of Bioinformatics (SIB), ETH Zürich, Basel, Switzerland

Thank you for addressing my previous comments for version 1 of this manuscript. I have no further comments.

Is the work clearly and accurately presented and does it cite the current literature?

Yes

If applicable, is the statistical analysis and its interpretation appropriate?

Partly

Are all the source data underlying the results available to ensure full reproducibility?

Yes

Is the study design appropriate and is the work technically sound?

Yes

Are the conclusions drawn adequately supported by the results?

Yes

Are sufficient details of methods and analysis provided to allow replication by others?

Yes

Reviewer Expertise:

Diagnostics; statistics

I confirm that I have read this submission and believe that I have an appropriate level of expertise to confirm that it is of an acceptable scientific standard.

# Peer review: Reviewer response for version 2

**DOI:** 10.21956/wellcomeopenres.19842.r50786

**Published:** 2022-05-27

**Authors:** Ankur Gupta-Wright

**Affiliations:** 1 Institute for Global Health, University College London, London, UK

I'm happy with the revisions, I have no further comments.

Is the work clearly and accurately presented and does it cite the current literature?

Yes

If applicable, is the statistical analysis and its interpretation appropriate?

Yes

Are all the source data underlying the results available to ensure full reproducibility?

Yes

Is the study design appropriate and is the work technically sound?

Yes

Are the conclusions drawn adequately supported by the results?

Yes

Are sufficient details of methods and analysis provided to allow replication by others?

Yes

Reviewer Expertise:

Diagnostics, Infectious Diseases

I confirm that I have read this submission and believe that I have an appropriate level of expertise to confirm that it is of an acceptable scientific standard.

# Peer review: Reviewer response for version 1

**DOI:** 10.21956/wellcomeopenres.19045.r48522

**Published:** 2022-02-11

**Authors:** Hans-Michael Kaltenbach

**Affiliations:** 1 Department of Biosystems Science and Engineering, Swiss Institute of Bioinformatics (SIB), ETH Zürich, Basel, Switzerland

This study determines the diagnostic accuracy of the Fortress LFIA for SARS-CoV-2 IgG antibodies after vaccination against an established reference assay. Specificity and sensitivity are estimated from samples of two cohorts and pooled estimates are reported as the main result. The authors provide an in-depth analysis of false-positive results and report additional results of neutralisation assays for one cohort. The results show lower sensitivity and higher specificity compared to the two cited previous evaluations of this LFIA, but both differences are not statistically significant. Overall, the study is well documented and the availability of raw data allows easy reproduction of the results.

 My only concern is the sample design and analysis of the HCW cohort. The calculations assume that the 78 samples are independent, but they are in fact pairs from 39 individuals measured before and after vaccination. I would sympathize with neglecting this issue for cases with a negative result at day 0 followed by a positive result at day 21, which one might treat as ‘sufficiently’ independent; however, one is not surprised to find the 13 positive samples of day 0 to also test positive at day 21. An ad-hoc remedy for this double-counting is to remove 13 of these 26 samples from the calculation as they provide no new information and artificially inflate the sample size. The estimated sensitivity is then reduced to 87.7% with a wider 95%-CI of [75.8, 97.1] (compare to the reported 92.3%) for that cohort, and to 91.0% (95%-CI [84.1, 95.6]) instead of 92.0% for the pooled analysis. Specificities are of course unaffected. The equally problematic case of negative results on both occasions does not occur in these data. Please comment on the sample design of the HCW cohort and its consequences.

 Three minor corrections:

- A significance level of 15% is reported for the sample size calculation; statistical folklore and the reported sample size suggest it to mean 5%.
- In Table 2, you report N=39 for the D21 seropositive column, but males and females add to 40.
- Please add a label for the vertical axis of Figure 1.

Is the work clearly and accurately presented and does it cite the current literature?

Yes

If applicable, is the statistical analysis and its interpretation appropriate?

Partly

Are all the source data underlying the results available to ensure full reproducibility?

Yes

Is the study design appropriate and is the work technically sound?

Yes

Are the conclusions drawn adequately supported by the results?

Yes

Are sufficient details of methods and analysis provided to allow replication by others?

Yes

Reviewer Expertise:

Diagnostics; statistics

I confirm that I have read this submission and believe that I have an appropriate level of expertise to confirm that it is of an acceptable scientific standard, however I have significant reservations, as outlined above.

# Response

**Published:** 2022-05-21

**Authors:** Alexandra Cann

**Affiliations:** Imperial College London, UK

My only concern is the sample design and analysis of the HCW cohort. The calculations assume that the 78 samples are independent, but they are in fact pairs from 39 individuals measured before and after vaccination. I would sympathize with neglecting this issue for cases with a negative result at day 0 followed by a positive result at day 21, which one might treat as ‘sufficiently’ independent; however, one is not surprised to find the 13 positive samples of day 0 to also test positive at day 21. An ad-hoc remedy for this double-counting is to remove 13 of these 26 samples from the calculation as they provide no new information and artificially inflate the sample size. The estimated sensitivity is then reduced to 87.7% with a wider 95%-CI of [75.8, 97.1] (compare to the reported 92.3%) for that cohort, and to 91.0% (95%-CI [84.1, 95.6]) instead of 92.0% for the pooled analysis. Specificities are of course unaffected. The equally problematic case of negative results on both occasions does not occur in these data. Please comment on the sample design of the HCW cohort and its consequences. 

- *Many thanks for this comment. We acknowledge as a result of the pragmatic study design, some participants in the HCW cohort (13) have provided two samples to the study, at different time points (>21 days apart), which have been treated as independent samples for the purpose of testing the accuracy of the LFIA devices against gold-standard. We agree that if this study was looking at individual differences in participants, it would not be appropriate to include both sets of samples as this would introduce bias. In individuals who have provided two samples, it is likely that both the quantitative and qualitative immune responses will be different. As such, given that the primary outcome for this study is to test the diagnostic accuracy of the LFIA device against the gold-standard Abbott titre we feel it is helpful to include these samples in the analysis. *
*Acknowledging the reviewer's comment, we have included a section addressing this in the discussion section and have shown the proposed sensitivity analysis removing the duplicate participants' samples’ in the supplement.*

A significance level of 15% is reported for the sample size calculation; statistical folklore and the reported sample size suggest it to mean 5%.

- *This was a mistake and has been edited in the paper. The significance level is 5%.*

In Table 2, you report N=39 for the D21 seropositive column, but males and females add to 40. 

- *This has been changed.*

Please add a label for the vertical axis of Figure 1.  

- *Label added.*

# Peer review: Reviewer response for version 1

**DOI:** 10.21956/wellcomeopenres.19045.r47744

**Published:** 2022-02-01

**Authors:** Ankur Gupta-Wright

**Affiliations:** 1 Institute for Global Health, University College London, London, UK

This study assesses the diagnostic accuracy of the Fortress LFIA for SARS-CoV-2 anti-S Ab compared to a lab-based serological assay reference standard. The study is appropriately conducted, based on two different cohorts but giving similar results. The authors have attempted to understand if FP results are due to FN results from the reference standard by using an additional assay for discordant results.

 Comments that need addressing:

Discussion

- The small sample size leads to wide CIs - this needs to be emphasised in the discussion/limitations section.
- Please state what the previously reported diagnostic accuracy for natural infection was for this assay.

Is the work clearly and accurately presented and does it cite the current literature?

Yes

If applicable, is the statistical analysis and its interpretation appropriate?

Yes

Are all the source data underlying the results available to ensure full reproducibility?

Yes

Is the study design appropriate and is the work technically sound?

Yes

Are the conclusions drawn adequately supported by the results?

Yes

Are sufficient details of methods and analysis provided to allow replication by others?

Yes

Reviewer Expertise:

Diagnostics, Infectious Diseases

I confirm that I have read this submission and believe that I have an appropriate level of expertise to confirm that it is of an acceptable scientific standard.

# Response

**Published:** 2022-05-21

**Authors:** Alexandra Cann

**Affiliations:** Imperial College London, UK

The small sample size leads to wide CIs - this needs to be emphasised in the discussion/limitations section.

- *Thank you for this comment. We have addressed this in the discussion/limitations section.*

Please state what the previously reported diagnostic accuracy for natural infection was for this assay.

- *We have included information and the reference paper on the reported diagnostic accuracy for natural infection for this assay.*
